# Evaluation of the Oxford Hip Score: Does it still have content validity? Interviews of total hip arthroplasty patients

**DOI:** 10.1186/s12955-021-01869-8

**Published:** 2021-10-09

**Authors:** Camilla Holmenlund, Søren Overgaard, Randi Bilberg, Claus Varnum

**Affiliations:** 1 Department of Orthopaedic Surgery, Lillebaelt Hospital - Vejle, University Hospital of Southern Denmark, Odense, Denmark; 2 Department of Orthopaedic Surgery and Traumatology, Odense University Hospital, Odense, Denmark; 3 OPEN, Odense Patient Data Explorative Network, Odense University Hospital, J.B.Winsløws Vej 4 Ground Floor, Entrance 9, 5000 Odense, Denmark; 4 Department of Clinical Research, Faculty of Health Sciences, University of Southern Denmark, Odense, Denmark; 5 Department of Orthopaedic Surgery and Traumatology, Copenhagen University Hospital, Bispebjerg, Bispebjerg Bakke 23, 2400 København NV, Denmark; 6 Department of Clinical Medicine, Faculty of Health and Medical Sciences, University of Copenhagen, Copenhagen, Denmark; 7 Department of Clinical Research, Unit of Clinical Alcohol Research, University of Southern Denmark, J.B. Winsløws vej 18, entrance 220B, 5000 Odense, Denmark; 8 Department of Regional Health Research, University of Southern Denmark, Beriderbakken 4, 7100 Vejle, Denmark

**Keywords:** Patient-reported outcome measures, Oxford Hip Score, Total hip arthroplasty, Focus group interviews, Content validity, Qualitative study

## Abstract

**Background:**

The Oxford Hip Score is used to evaluate the outcome after total hip arthroplasty. The Oxford Hip Score was developed more than 20 years ago with only some degree of patient involvement. We question if the Oxford Hip Score is still relevant for the present-day total hip artrhoplasty patients. We aimed to determine whether the Oxford Hip Score contains items that are relevant for present-day patients with osteoarthritis undergoing total hip arthroplasty, thus investigating the content validity.

**Methods:**

Patients aged 60–75 years, undergoing total hip arthroplasty for primary osteoarthritis were recruited to participate in focus group interviews preoperatively and at 3 and 12 months after primary total hip arthroplasty. We conducted 6 focus group interviews in which 30 patients participated. The interviews were audio-recorded and transcribed verbatim. Using Interpretative Phenomenological Analysis, we inductively organised the interview transcripts into particular items/themes which we then compared to items in the Oxford Hip Score.

**Results:**

We identified 6 general items with 41 sub-items. The 6 general items were pain, walking, physical activities, functional abilities, quality of life and psychological health. We found that items in the Oxford Hip Score were all in some way relevant to the patients but that the Oxford Hip Score lacks several important items relevant for present-day total hip artrhoplasty patients, including several physical activities, functional abilities and certain aspects of quality of life and psychological health.

**Conclusion:**

We found that the Oxford Hip Score lacks important items for present-day patients in our population. Due to findings regarding several additional items that are not present in the Oxford Hip Score, particularly concerning physical activities and quality of life, we question the content validity of the Oxford Hip Score for a present-day population. Our findings indicate a need for a revision of the Oxford Hip Score.

## Introduction

Patient-reported outcome measures (PROMs) are important in the evaluation of patient outcome and quality of life following total hip arthroplasty (THA) [1, 2]. Hip-specific PROMs primarily focus on patients’ level of pain and joint stiffness and functioning in typical daily activities [3, 4]. Patients should be included in the development of PROMs to ensure content validity and, thereby, reliability, thus reducing the ceiling effect [5, 6]. Only when a PROM truly represents the perspectives of patients it differs from the traditional objective evaluation methods [7].

In this study, we opted to focus on the Oxford Hips Score (OHS) as it is used in research worldwide [8, 9], is included in national databases [8, 10] and has been used and validated in several countries [4, 11, 12]. The OHS, published in 1996 [4], was developed as a joint-specific PROM and aimed to be a shorter version with a higher level of responsiveness than other PROMs at that time.

The content of the OHS was developed by a research team that interviewed 20 patients and reviewed established questionnaires resulting in a list of 20 items. Through modification and retesting, this list ultimately morphed into a 12-item questionnaire assessing pain and function of the hip (Table 1) [4].

**Table 1:** The Oxford Hip Score

During the past four weeks …	(Tick one box for each question)				
1. How would you describe the pain you usually have from your hip?	None	Very mild	Mild	Moderate	Severe
	□	□	□	□	□
2. Have you had any trouble with washing and drying yourself (all over) because of your hip?	No trouble at all	Very little trouble	Moderate trouble	Extreme difficulty	Impossible to do
	□	□	□	□	□
3. Have you had any trouble getting in and out of a car or using public transport because of your hip?	No trouble at all	Very little trouble	Moderate trouble	Extreme difficulty	Impossible to do
	□	□	□	□	□
4. Have you been able to put on a pair of socks, stockings, or tights?	Yes, easily	With little difficulty	With moderate difficulty	With extreme difficulty	No, impossible
	□	□	□	□	□
5. Could you do the household shopping on your own?	Yes, easily	With little difficulty	With moderate difficulty	With extreme difficulty	No, impossible
	□	□	□	□	□
6. For how long have you been able to walk before pain from your hip becomes severe? (With or without a walking aid)	No pain/more than 30 min	16 to 30 min	5 to 15 min	Around the house only	Not at all—pain severe on walking
	□	□	□	□	□
7. Have you been able to climb a flight of stairs?	Yes, easily	With little difficulty	With moderate difficulty	With extreme difficulty	No, impossible
	□	□	□	□	□
8. After a meal (sat at a table), how painful has it been for you to stand up from a chair because of your hip?	Not at all painful	Slightly painful	Moderately painful	Very painful	Unbearable
	□	□	□	□	□
9. Have you been limping when walking because of your hip?	Rarely/never	Sometimes or just at first	Often, not just at first	Most of the time	All of the time
	□	□	□	□	□
10. Have you had any sudden, severe pain—'shooting', 'stabbing' or 'spasms' from your affected hip?	No days	Only 1 or 2 days	Some days	Most days	Every day
	□	□	□	□	□
11. How much has the pain from your hip interfered with your usual work, including housework?	Not at all	A little bit	Moderately	Greatly	Totally
	□	□	□	□	□
12. Have you been troubled by pain from your hip in bed at night?	No nights	Only 1 or 2 nights	Some nights	Most nights	Every night
	□	□	□	□	□

A validated Danish translation was used in this study with the design presented in this table

Studies that have compared the OHS with other PROMs suggest that the OHS has some degree of ceiling effect [13–15] which could indicate that items are missing for patients with high scores, thereby limiting the content validity [5, 16]. The reduced content validity may be due to the higher expectations of present-day THA patients. Furthermore, the improvement in THA treatments produces high levels of function [17, 18] which could have further affected patients’ expectations. Therefore, it is important to re-evaluate the content validity of the OHS.

We aimed to investigate the content validity of the OHS by determining whether the OHS contains items relevant for present-day THA patients.

## Patients and methods

### Design and setting

We adopted a qualitative approach based on focus group interviews to explore content validity by gaining insight into patient’s experiences of THA surgery. The qualitative approach yields data concerning our research question not obtainable by quantitative research methods [19, 20].

Recognising that the case-mix of patients would likely differ between a university and a regional hospital, we recruited patients equally from Odense University Hospital and Lillebaelt Hospital, Vejle. This was done to obtain more diversity in the interview groups and thereby greater generalizability.

### Recruiting patients

We aimed to recruit 6 patients for each focus group interview as we assessed this was the ideal focus group size, with the presumption that patients would be highly emotionally involved in and experts on the topic [21]. We conducted 6 focus group interviews in total to achieve theoretical data saturation [21–23]. Interviews were conducted before surgery and at 3 and 12 months after surgery as these are relevant intervals in the patient pathway and are used in registers [8]. We planned two focus group interviews at each time point, holding one interview at each hospital for each time point. We aimed for an even distribution of gender in each focus group. To obtain information about the situation of the patients before and 3 and 12 months after surgery, we recruited different patients who were currently being treated at these time points. The patients did not participate twice.

A heterogeneous sample of THA patients fulfilling the inclusion criteria was purposively sampled by gender, location and time point. The inclusion criteria were patients aged 60–75 years and diagnosed with primary osteoarthritis, and patients interviewed at 3 and 12 months postoperatively should have THA performed through a posterolateral approach. We excluded patients who presented with symptoms elsewhere in the lower extremities or lower back not distinguishable from the affected hip and patients whose walking was affected by a THA of the opposite hip. Furthermore, patients were excluded if they could not speak, read or understand Danish or were mentally unable to participate.

**Fig. 1:**
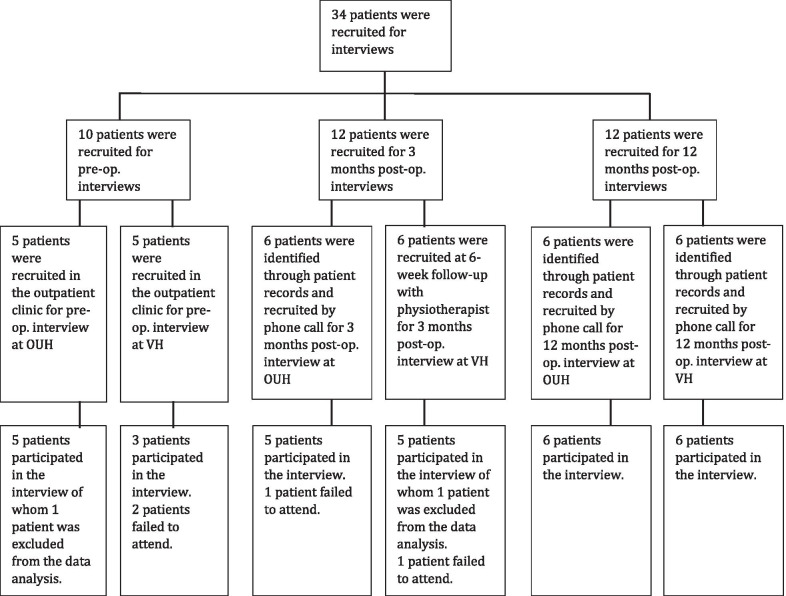
Diagram showing the recruiting of patients. Patients excluded from data analysis did not fulfil the inclusion criteria. OUH, Odense University Hospital; VH, Vejle Hospital

As the recruiting period was characterised by fewer surgeries than expected, only 5 patients were recruited for each of the two preoperative interviews. In total, we recruited 34 patients, 17 men and 17 women, for 6 focus group interviews which were conducted between October 1, 2017, and April 30, 2018 (Fig. 1).

10 patients were recruited in the clinic while being registered for primary THA for preoperative interviews. 6 patients were also recruited in the clinic during their 6-week follow-up visit with a physiotherapist for the 3 months postoperative interview at Vejle Hospital. 6 patients for the 3 months postoperative interview at Odense University Hospital and 12 patients for both the 12 months postoperative interview were found from patient records and invited to attend by phone call.

4 patients failed to attend the interviews and 2 patients did not meet the inclusion criteria which led to the exclusion of their data from the data analysis (Fig. 1). Data from 28 patients were used in the analysis.

### Focus group interviews

**Fig. 2:**
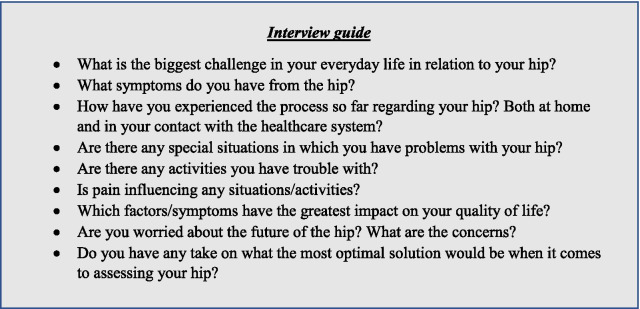
The interview guide used in the focus group interviews

We developed a semi-structured interview guide with 8 open-ended questions (Fig. 2). All questions were exploratory to enable differences between patients’ opinions to emerge during the interviews. Follow-up questions during the interviews were not predetermined but were formed according to the dynamics of the conversation. To confirm the length of the interviews (approximately 1 to 1.5 h in duration) and ensure questions were clear and comprehensible, we tested the interview guide in a pilot interview with 6 preoperative patients.

The interviews were conducted by CH, a pre-graduate medical student with knowledge from the literature and with the endorsement of RB, a postdoctoral qualitative researcher. The interviewer had no previous relationship with the patients. Signed consent was obtained from the patients before the interviews.

We did not disclose which PROM was under investigation as it was not the focus of this study to obtain opinions on the PROM. During the interviews, we encouraged patients to speak freely and emphasised that there were no right or wrong answers. We conducted focus group interviews to obtain a wide array of perspectives that emerge in the context of group discussion. We did not include the interaction between the focus group participants as we aimed to analyse their answers according to the OHS.

All focus group interviews were audio-taped and a verbatim transcription was supported by NVivo [24]. The patients were anonymised in the transcripts.

### Analysis of findings

For analyses of the transcripts, we used the Interpretative Phenomenological Analysis (IPA) [25]. IPA is a phenomenological approach analysing the patient’s experiences of, in this case, their upcoming THA or at 3 and 12 months after surgery. The hermeneutic approach of IPA makes it possible to compare the patients’ experiences with the OHS. To use IPA, a limited number of participants were included in each focus group interview to ensure the thorough investigation of each participant’s experience and statement included in the interview [25]. We read and reread the transcripts to identify all patient statements regarding their hip and then coded all statements. Subsequently, we identified main themes/items from the coding [26].

## Results

### Demographic characteristics

The total sample of interview patients consisted of 13 (46%) women and 15 (54%) men with a mean age of 70 years and there was no significant difference in the distribution of gender or age between the periods or hospitals (Table 2).

**Table 2:** Characteristics of the patients participating in the interviews

	Interviews		
	Pre-op	3 mths. post-op	12 mths. post-op
Hospital			
OUH (n (%))	4 (57%)	5 (56%)	6 (50%)
VH (n (%))	3 (43%)	4 (44%)	6 (50%)
Age			
Years (Mean (SD))	68 (5)	70 (2)	71 (4)
Years (Range)	60–72	66–73	64–75
Gender			
Male (n(%))	4 (57%)	5 (56%)	6 (50%)
Female (n%))	3 (43%)	4 (44%)	6 (50%)

SD, standard deviation; OUH, Odense University Hospital; VH, Vejle Hospital; mths., months; Pre-op., preoperative; Post-op., postoperative

### Overall findings

From the analysis of the transcripts, we identified 6 general and 41 sub-items. The 6 general items were pain, walking, physical activities, functional abilities, quality of life and psychological health (Table 3). By comparing these items to the OHS we found that the items on the OHS were all relevant to the patients, although some items were more predominant than others. However, we found several sub-items related to pain and walking, physical activities, functional abilities and some aspects of quality of life and psychological health that the OHS does not cover.

**Table 3:** Table of items identified from the inductive content analysis

Main item	General items	Sub-items
Patient views on everyday life when undergoing total hip arthroplasty	Pain	Pain in general
		Nightly pain
		Use of pain medication
		Daily variation in pain
		Diverted pain caused by the hip
		Repayment of pain after activities
		Sudden pain episodes
		Stiffness/mobility of the leg
	Walking	Walking distance
		Walking pace
		Uneven terrain
		Walking aid
		Limping
	Physical activities	Cycling
		Running
		Climbing ladders
		Sport (fitness, table tennis, tennis, badminton, soccer, golf, dancing, gymnastics, skiing, hunting)
		Lifting heavy objects
	Functional abilities	Bathing
		Using the car
		Dressing (especially regarding socks)
		Shopping
		Ascending/descending stairs
		Sitting/standing from a chair
		Housework incl. gardening
		Standing
		Lower one-self onto the floor/getting up from the floor
		Starting difficulty (after sitting, driving, cycling)
		Lying on the operated hip (hard surface)
	Quality of life	Affecting social life/vacations
		Slower pace than normal/the need to rest
		Using helping aids/making changes to the house
		Performing activities that improve individuals’ quality of life
	Psychological health	Feels like a burden to others
		Falling
		Overloading the opposite hip
		Dislocating the hip/thinks about positioning the body
		Survival of the hip
		Difference in leg length
		Awareness of the hip
		Cold weather

#### Pain

Pain is covered by the OHS as the description of pain, the experience of sudden pain and nightly pain. Many of the patients indicated pain as one of the greatest challenges and described pain relief as a spectacular feeling.

- *‘The most important thing was to achieve pain relief… And after surgery, when you discover that you are completely pain free, it is fantastic’* (Male, 69 years, 12 months postoperatively).

Patients also described uncomfortable episodes of sudden shooting pain and how pain affected their sleep. The patients described how being awakened by their pain at night was both disturbing and disruptive to their daily lives due to resulting exhaustion.

- *‘For me, the biggest challenge has been being able to sleep well at night. Because getting your sleep at night, for my part, has meant that I get 1–2 h extra per day’* (Male, 66 years, 12 months postoperatively).

The patients also focused on other sub-items related to pain not covered by the OHS, including the use of pain medication and repayment of pain following activity. The patients wished to underline the weak correlation between the intake of pain medication and the extent to which they experienced pain. They attributed great importance to the pain medication item.

- *‘The physician could see that the hip was severely damaged, he just couldn’t understand that I was still able to run, or at least I did despite the pain and I didn’t take any pain medication even though I was in pain’* (Male, 66 years, 12 months postoperatively).

#### Walking

The patients discussed many aspects of walking ability and there was a consensus that this was a significant factor of THA where they experienced problems, for example, no longer being able to walk the desired distance and limping due to pain.

- *‘The fact is that I really want to go for a walk but when I am done walking… then I am completely worn out when I get home’* (Female, 71 years, 12 months postoperatively).

This aspect of walking is covered by the OHS. However, we also found aspects of walking such as walking pace and walking on uneven terrain that the OHS does not cover. These aspects also affect the patients’ quality of life as they feel unable to keep pace with their relatives or walk on the beach when on holiday.

#### Physical activities

Sub-items that demanded more physical exertion than that required to complete everyday functions were categorised as physical activities. Physical activities are not covered by the OHS, which focuses more on everyday activities. We found that patients have fairly high demands regarding their level of physical activities. For example, they place great importance on their ability to participate in cycling, running, sport and lifting heavy objects; the most predominantly important activities were cycling and sports.

The ability to ride a bike was described as important both as a method of transportation and a form of gentle exercise.

- *‘When I ride a bike, I feel that there isn’t the same mobility in the hip that there used to be. I used to cycle a lot’* (Female, 66 years, preoperatively).

The patients also emphasised the importance of the ability to participate in their preferred sports and several described sports as activities that are part of a healthy social life, thereby enhancing their quality of life.

- *‘But it is just because there is so much social connected to it. You get around and play with and are together with the same people’* (Male, 62 years, preoperatively).

#### Functional abilities

Several everyday functions are covered by the OHS: bathing, putting on socks, shopping, using the car, climbing stairs, completing household tasks and getting up from a chair. These were all mentioned during the interviews, with some being more predominant, such as climbing stairs and putting on socks.

- *‘I have a daughter who lives on the 4th floor, and I keep myself from visiting her because the stairs are a nuisance for me. Alternatively, I must take pain medication before I go, and then it sort of works when I get there, and then I can climb the stairs’* (Male, 67 years, preoperatively).

However, we found that the patients’ demands for functional abilities went beyond those covered by the OHS. They included sitting down, standing up, lying on the operated site and, most predominantly, the ability to lower one-self onto and get up from the floor.

Patients’ ability to lower themselves to the floor to retrieve fallen objects was found to represent an important everyday activity. For several patients, this function was linked to the ability to play with their grandchildren.

- *‘For many years I haven’t been able to get on the floor and up again without many strange movements and maybe a helping hand, but I can do that now, so that is huge progress for me. I think it matters greatly, particularly when you have small children that you want to play with…’* (Female, 73 years, 3 months postoperatively).

#### Quality of life

Quality of life was very important for the patients we interviewed, yet, this is a category not covered by the OHS. The ability to engage in activities that enhance the individuals’ quality of life was emphasised as very important and the description of such activities illustrated that quality of life concerns more than the ability to accomplish typical daily activities.

- *‘Quality of life, for me, is being able to walk on the beach and climbing stairs effortlessly and being able to walk … So that it is kind of the parameter for … Well cleaning and vacuuming is something I could buy myself out of, if it had to come to that, but the other things are quality of life to me’* (Female, 68 years, 12 months postoperatively).

The patients also described how the pain was affecting social life and holidays:

- *‘And then again, as you say, the social stuff. The fact that you are forced to be the one that sets the limitations all the time or staying home instead… Also, in relation to the pain, it makes me mad and less happy than I used to be’* (Female, 66 years, preoperatively).

#### Psychological health

Psychological health, which could be considered an extension of quality of life, is also not covered by the OHS. Though many of the sub-items related to psychological health were not predominant, they were mentioned during the interviews and included fear of falling and/or dislocating the hip and awareness of the hip.

A more predominant theme regarding psychological health was the feeling of being a burden to others.

- *‘It's so tiring to be the one to put a stop to things’* (Female, 66 years, preoperatively).

## Discussion

This study investigated the content validity of the OHS using a qualitative approach.

We found that there are several additional items that present-day patients deem relevant which are not included in the OHS. This indicates that the OHS fails to cover all the important items regarding living with an artificial hip, which questions the content validity of the OHS.

There are limited studies investigating the patients’ perspectives on the OHS. Wylde et al. [27] allowed patients to make annotations when administering the OHS; 5 items were heavily annotated. One annotation on the subject of pain was ‘it depends on pain medication’. Other items were frequently annotated with ‘due to other co-morbidities’. McMurray et al. [28] administered the OHS before conducting semi-structured interviews interrogating difficulties encountered by patients. McMurray et al. [28] found that patients addressed problems as lacking specificity to the questions, clarity about the response category and difficulty with distinguishing the hip from other comorbidities. Furthermore, they also found questions surrounding pain were difficult for patients to answer due to their use of pain medication and their ability to adapt to life with pain. Both of these studies question the validity of the OHS [27, 28]. Our study also found that patients placed much emphasis on pain medication and discussed several additional items not included in the OHS, supporting the questionability of the content validity of the OHS regarding pain.

A Dutch translation and validation of the OHS from Gosens et al. [12] attempted to incorporate items such as ‘the need for walking aids’ and ‘sexual problems caused by the hip’ based on experience with hip patients. Gosens et al. [12] found these to be important items for which many patients had a low score, however, some patients declined to answer the item ‘sexual problems caused by the hip’, which could be problematic due to lowering the consistency of the findings. Our study also identified the item of ‘the need for walking aids’, however, none of the patients in the present study alluded to sexual problems, which may be attributable to the focus group setting and the sensitive nature of the subject.

The group of researchers who developed the OHS published a review of the OHS in 2007 [8]. In contrast to our findings, they asserted that an extension of the OHS incorporating items identified by Gosens et al. [12], such as ‘the use of walking aids’ and ‘problems with sexual activity’, would not be beneficial. They justified this assertion because these issues were not found to be important in their initial development interview. Hence, they suggested that the issues deemed important and relevant by patients do not change over time. This is in contrast to our findings which suggest that current patient preferences are higher compared to those at the time of development of the OHS.

The findings of additional items in this study may be attributable to the decrease in physical limitations and increase in self-reported health [29, 30], as well as the increase in life expectancy that has emerged since the OHS was published [31, 32]. These developments enable the elderly to enjoy a more active and independent lifestyle, which may foster the formation of different expectations regarding quality of life after THA [33–35].

The development in lifestyle are illustrated in our findings as we found that quality of life ventures beyond the ability to accomplish typical daily activities and that patients expressed a need for a full and functioning social life. A study from Garbuz et al. [13] also highlighted that patient expectations are increasing, which the current OHS cannot meet, thereby suggesting the need for development of new outcome tools.

### Study strengths and limitations

The strengths of this study include the method of focus group interviews, which are conducive for studying the validity of a structured instrument and can provide insight regarding patients’ perspectives [19, 20]. By opting to recruit patients from both a university and a regional hospital, the influence of differences in, for instance, comorbidity among patients referred to a university hospital versus patients referred to a regional hospital was minimised, thereby increasing the generalisability of our findings. Further, we wanted to observe the issues specifically emphasised by the patients concerning their hips and therefore we deliberately avoided presenting the OHS to the patients before the interviews, thus yielding additional items beyond those covered by the OHS.

One author conducted all the interviews and performed reading and coding of the transcripts before discussing the results with the other authors. Due to the nature of this form of qualitative analysis, the interpretation of findings is likely affected in some way by this one author. We did, however, base the interviews on open-ended questions and transcribed verbatim all patient statements which were extracted without filter, limiting the subjectivity of the analysis.

The patients in our study did not mention sensitive topics such as sexual function, which could be a limitation of this study, due to its focus-group-interview structure. Further, it should be acknowledged that this study was conducted within the context of Danish culture, with items such as cycling being particularly popular among Danish communities. Our findings may not be generalisable to other cultures.

## Conclusion

This study showed that the OHS lacks important items for present-day patients in our population. Due to the findings of several additional important items, we question the content validity of the OHS in a present-day population.

The additional items identified in this study as relevant to patients suggest that present-day patients have high expectations of life with THA.

Considering the results obtained by previous studies [13, 27, 28] and in conjunction with our results, they may indicate a need for revision of the OHS so that it embraces all items relevant to THA patients. We recognize concerns regarding response rate of a lengthy PROM. There are, however, other factors that impact response rate, and previous studies have shown low correlations between response rate and questionnaire length [36, 37].

Thus, patient involvement is of the utmost importance throughout all the steps involved in the development and validation of a PROM to ensure content validity. As shown in this study, patient preferences may change over time and this calls into question the use of PROMs that have not undergone validation within recent years.

## Data Availability

The dataset supporting the conclusions of this article is available from the corresponding author on reasonable request.

## Abbreviations

PROMs: Patient-reported outcome measures
THA: Total hip arthroplasty
OHS: Oxford Hip Score
IPA: Interpretative Phenomenological Analysis
